# Simulation research on dynamic characteristics of scroll compressor with flexible clearance

**DOI:** 10.1371/journal.pone.0302711

**Published:** 2025-02-03

**Authors:** Haifei Qiu, Zhang Yifan, Li Feiyang, Chen Xiandong

**Affiliations:** 1 School of Mechanical Engineering of XIJING University, Xi’an, China; 2 Engineering Research Center of Hydrogen Energy Equipment & Safety Detection, Universities of Shaanxi Province, Xijing University, Xi'an, China; Monash University Malaysia, MALAYSIA

## Abstract

The scroll compressor is a rotating machinery with high-speed and high-precision characteristics. In this paper, a vector model of the kinematic pair was constructed based on two-state clearance. To confirm the adverse effects of tiny clearance (0<r≤0.1mm) on the scroll compressor, a dynamics simulation of the spindle was performed under flexible contact conditions with different clearances. The study indicates that simulation results for flexible and rigid bodies are vastly different, and the former is closer to reality. Compared with the case in an ideal state (0mm), clearance at 0.01mm and 0.04mm can cause an enormous impact on the critical bearing components of the spindle, such as the orbiting scroll, cross-slip ring, and support bearing, which results in unexpected vibration and noise. However, when the clearance increases to 0.07mm and 0.1mm, the collision amplitude and friction resistance inside the needle bearing are relatively small, which avoids violent impacts on the machine. This paper considers the effect of spindle flexibility and transmission clearance simultaneously, which helps to explore the running mechanism of a scroll compressor with clearance.

## Introduction

The scroll compressor is a lightweight, small, efficient gas compression machine that produces low vibration and noise. It is widely used in various fields such as transportation, power engineering, air conditioning, refrigeration, the petrochemical industry, and more [1]. The manufacturing level of the scroll compressor has significantly improved in recent years due to the rapid development of numerical control processing techniques, and its working speed has been achieved at 12000 r/min. The spindle component is the heart of the scroll compressor, and its dynamic performance dramatically affects gas compression efficiency and quality [2]. Therefore, ensuring the spindle has excellent transmission accuracy and running stability is crucial to keep up with the scroll compressor’s high speed and high-efficiency characteristics.

In reality, any mechanical system faces inevitable transmission clearance due to assembly, manufacturing, friction, and wear errors. The ideal state is to have a kinematic pair without clearance. Studies have shown that when a scroll compressor’s spindle component runs ultra-speed, a kinematic pair with clearance causes contact distortion, high-frequency collisions, and nonlinear impact loads. As a result, the actual rotation track of the spindle deviates from its ideal track and may even cross it [3]. It is beyond doubt that running for an extended period under these conditions will weaken the load transmission of the spindle system, thereby reducing its working reliability and may even result in damage and failure of the joints, leading to unpredictable mechanical failures or the need for outage maintenance.

Several universities and enterprises, such as Lanzhou University of Technology, Jiangxi University of Science and Technology, Northern Navigation and Control Technology Co. Ltd., and Tianjin Danfoss Co. Ltd., have made significant progress in clearance research on the scroll compressor. They have conducted various studies on the compressor, including rotor dynamic modeling and simulation to exhibit the contact force and motion parameters of the kinematic pair under different clearances, numerical simulation of the flow field in the compressor’s inner cavity under different radial clearance to respond to the leakage; and dynamic simulation on the small shaft anti-rotation mechanism, clearance size, and clearance number to provide reference for reasonable selection of bearing clearance. Additionally, some researchers have used the Monte Carlo method to simulate and analyze the spindle axial clearance to optimize the deviation of clearance size [4, 5].

However, most previous studies on scroll compressors have only focused on their rigid body dynamics, neglecting the spindle system’s elastic deformation and stress distribution. This approach fails to accurately reflect the micro-amplitude vibration and small deformation caused by joint clearance, which reduces scroll compressors’ high-speed and high-precision design levels.

Flexible modeling techniques are commonly used to analyze components’ small vibrations and deformations under high-speed conditions. Compared to rigid structures, flexible bodies can more accurately represent the kinematic and dynamic characteristics of mechanisms, significantly improving simulation analysis’s accuracy and reliability [6]. Flexible modeling replaces the original rigid body with a structural discretization technique. The basic idea is to represent the elastic displacement of the object through a linear combination of modal vectors and modal coordinates, which approximates the infinite degrees of freedom of the actual structure through a finite number of element node degrees of freedom of the flexible body [7]. In a word, flexible modeling of the mechanism helps approximate objects’ proper motion form more accurately, which improves simulation accuracy and reduces errors.

The main focus of this paper is to study the dynamic modeling and simulation of the spindle system of a horizontal scroll compressor with a flexible clearance. The paper achieves this by combining theoretical analysis and mechanism design to produce various analysis results and simulation data. These results aid in understanding the working state of scroll compressors with clearance. The research findings can be of significant reference value for the performance evaluation of scroll compressors with clearance, which is also the main feature of our work.

## Structure description

**Fig 1:**
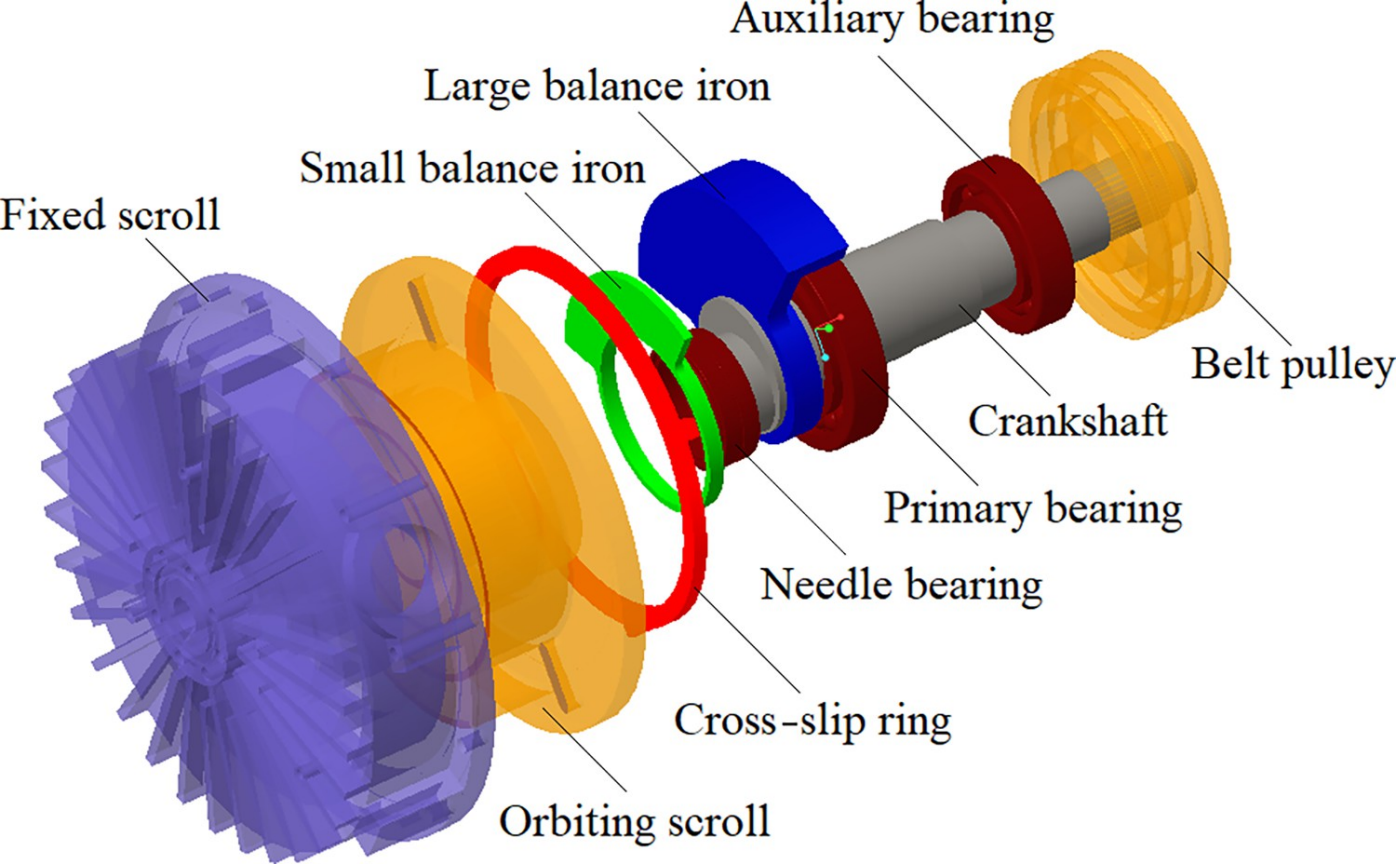
Spindle structure decomposition.

The scroll compressor inhales, compresses, and emits gas by changing the closed volume. Fig 1 illustrates that the spindle is designed with an eccentric structure that forms a closed volume cavity between the orbiting and fixed scrolls [8]. The crankshaft is fixed to the frame by supporting primary and auxiliary bearings. The fixed scroll is always static as it is rigidly fixed to the frame. The spindle is equipped with large and small balance irons in different locations to eliminate the influence of inertial centrifugal force generated by the crankshaft and the orbiting scroll. Moreover, a cross-slip ring is placed between the frame and the orbiting scroll to prevent the latter from rotating.

The running scroll compressor uses a belt pulley to transfer the motor torque to the eccentric crankshaft, which drives the orbiting scroll around the spindle system’s centerline. As the orbiting scroll rotates, it interacts periodically with the fixed scroll, creating several crescent-shaped cavities that compress and continuously change the gas inside the compressor. This process allows the compressor to inhale, compress, and emit external gas continuously. Table 1 shows this scroll compressor’s performance parameters, including *v*_max_-maximum speed, *q*-flow, *p*-exhaust pressure, *s*-compression ratio, *η*-volumetric efficiency, and *t*-exhaust temperature.

**Table 1:** Performance parameters of the scroll compressor.

*v*_max_/r·min^-1^	*q*/m^3^·min^-1^	*p*/MPa	s	η	*t*/°C
8500	≤500	≤320	3~5	98%	120~150

## Clearance vector model

In the spindle system, joint clearance can cause contact distortion, resulting in two states between the crank pin and the bearing: "free movement" and "contact deformation." Compared to other clearance models, the kinematic pair with two clearance states incorporates factors like elasticity, damping, and friction of moving components based on the nonlinear equivalent spring damping model [9]. Therefore, it can more accurately simulate the contact and collision mechanism of the rotary pairs. This work applies the two-state clearance model to design and analyze the bearing clearance.

**Fig 2:**
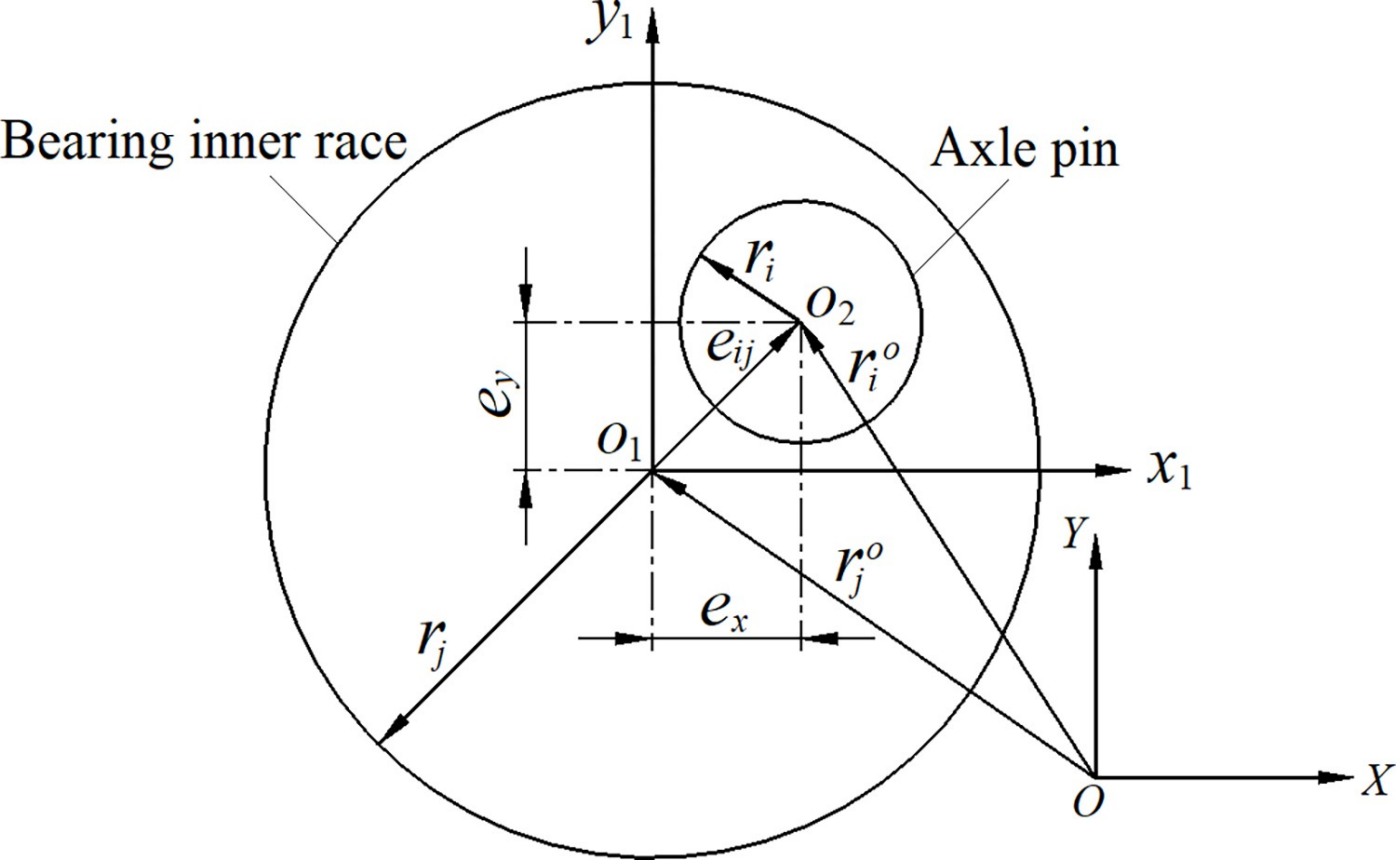
Clearance vector relationship.

In order to accurately describe the change in joint clearance, a vector *e*_*ij*_ is introduced into the radial section (*x*_1_*o*_1_*y*_1_) of the joint, as illustrated in Fig 2. The mathematical relationship of the clearance vector model between the motion of the axle pin and the bearing can be explained through Formula (1). Obviously, the clearance size *c* is a difference value between the bearing inner race radius *r*_*j*_ and the axle pin radius *r*_*i*_.


$$c=r_{j}-r_{i}$$ (1)

The clearance vector *e*_*ij*_ is defined in a local Cartesian coordinate system *x*_1_*o*_1_*y*_1_ and points from the bearing rotation center *o*_1_ to the crank pin rotation center *o*_2_ according to the composition of the generalized coordinate system *XOY*. These two coordinate systems correspond to the coordinate system of the scroll compressor spindle system, where both the *X* and *Y* axes and the *x*_1_ and *y*_1_ axes are located within the spindle’s radial section, while the *Z* and *z*_1_ axes perpendicular to them remain consistent with axis direction of the spindle. The Formula (2) represents the vector *e*_*ij*_ by analyzing geometric conditions.


$$e_{ij}=r_{i}^{o}-r_{j}^{o}$$ (2)

In the given formula, $r_{i}^{o}$ represents the position vector for the crank pin rotation center, while $r_{j}^{o}$ represents the position vector for the bearing rotation center in the generalized coordinate system. The variables *e*_*x*_ and *e*_*y*_ denote the center distance component of *o*_1_*o*_2_ in the *x*_1_ and *y*_1_ directions [10], respectively.

Following the vector model shown in Fig 2, the clearance vector *e*_*ij*_ can be determined by the Formula (3). Here, *e*_*x*_ denotes the component of the center distance *o*_1_*o*_2_ in the *x*_1_ direction, and *e*_*y*_ refers to the center distance *o*_1_*o*_2_ in the *y*_1_ direction.


$$e_{ij}={\left(e_{x}^{2}-e_{y}^{2}\right)}^{1/2}$$ (3)

During the rotation of a pair with clearance, it is inevitable that there will be contact collisions between the crank pin and the bearing. Formula (4) shows the calculating method of penetration depth (*δ*). It can be inferred that when *e*_*ij*_≥*c*, the rotating pair with clearance will be in contact while in a state of free detachment at *e*_*ij*_<*c* [11]. The position of the two rotation centers, *o*_1_ and *o*_2_, determines whether the crank pin comes into contact with the bearing.


$$\delta =e_{ij}-c$$ (4)

## Virtual prototype

### Kinematic constraints

**Fig 3:**
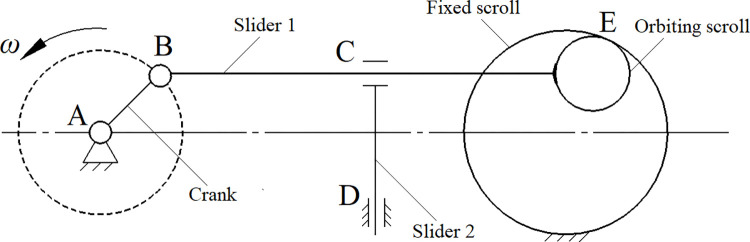
Transmission principle of scroll compressor.

In Fig 3, the scroll compressor is shown as a transmission mechanism that consists of one crank and two sliders [12]. As the crank rotates around point A, it drives sliders 1 and 2 simultaneously at point B, causing the orbiting scroll to engage with the fixed scroll at point E.

The orbiting scroll and the fixed scroll are connected to slider 1 and frame firmly, respectively, so the mechanism can be deemed a system with three moving components, four lower pairs (two rotating pairs and two translational pairs), and 0 higher pairs. According to the calculated method, as shown in Formula (5), the degree of freedom of the spindle system is *F* = 1, which satisfies the theory of mechanism. In the formula, variables *n*, *P*_L_, and *P*_H_ refer to the number of moving components, lower pairs, and high pairs, respectively.


$$F=3n-2P_{L}-P_{H}$$ (5)

**Fig 4:**
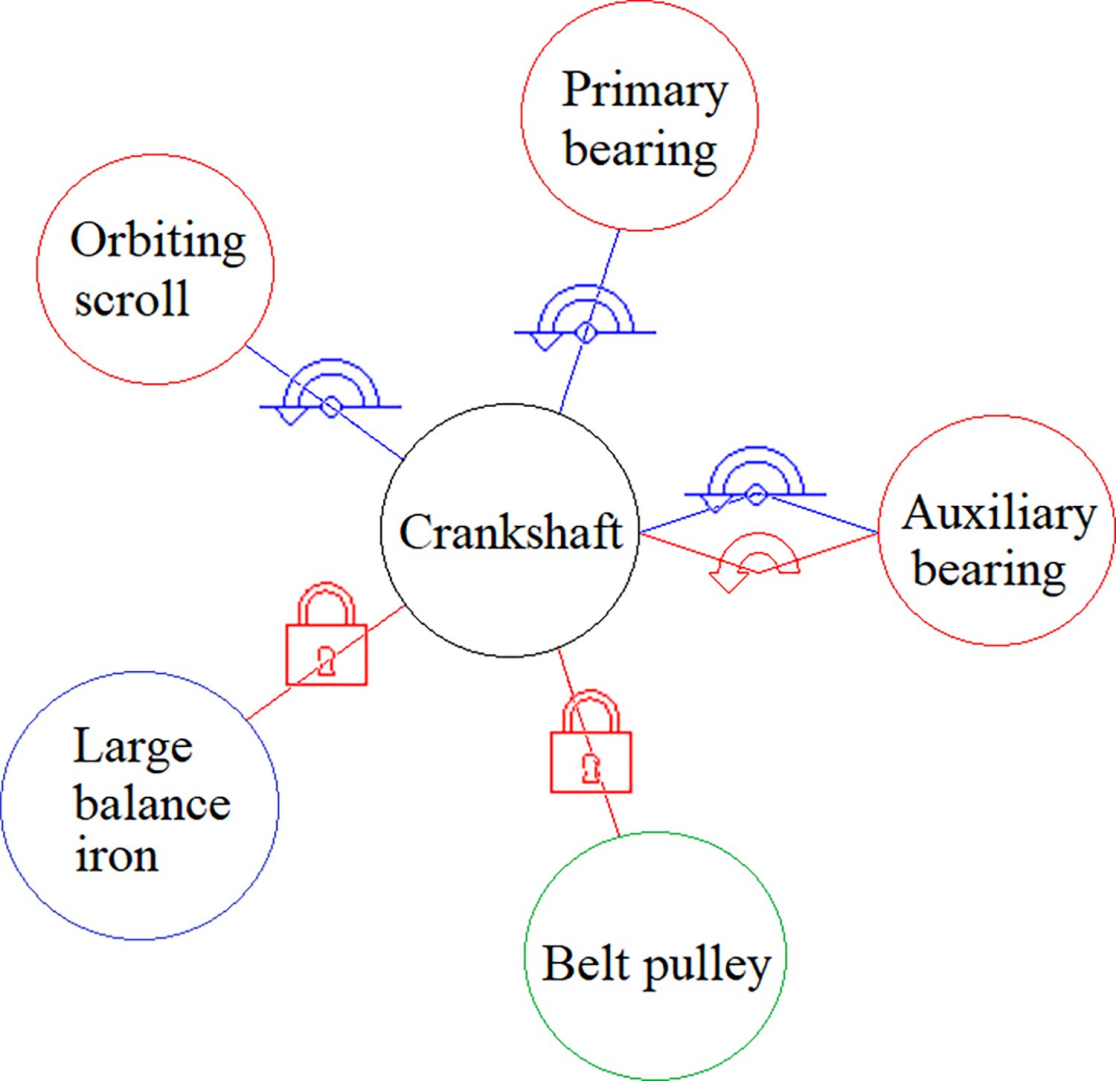
Kinematic constraint of the components.

A spindle simulation model was created using ADAMS/View software to examine the scroll compressor’s movement. The crankshaft, orbiting scroll, and primary and auxiliary bearings were subjected to constraints, as depicted in Fig 4. The motion drive of the rotating pair acted on the auxiliary bearing, while the crankshaft’s large balance iron and belt pulley were affixed with a fixed pair constraint. The small balance iron and cross-slip ring were fixed to the orbiting scroll and frame via a translational pair constraint. The *e*_*x*_ and *e*_*y*_ components represented the center distance of *o*_1_*o*_2_ in the *x*_1_ and *y*_1_ directions.

### Material properties

In line with the actual manufacturing process of the scroll compressor, several alloy materials such as Cr40, Cu2Cr2Mo, Cast iron, LY12, and HT200 are customized and added to the database of ADAMS/View, as displayed in Table 2 [13]. These alloy materials are then assigned to different simulation model components, including the crankshaft, orbiting scroll, fixed scroll, cross-slip ring, large and small balance irons, and belt pulley.

**Table 2:** Material properties of the spindle.

Component	Material	Mass/kg	Rotary inertia/kg·m^2^		
			I_xx_	I_yy_	I_zz_
Crankshaft	Cr40	5.31	3.31×10^−2^	3.30×10^−2^	2.28×10^−3^
Orbiting scroll	Cu_2_Cr_2_Mo	7.71	5.74×10^−2^	3.17×10^−2^	3.07×10^−2^
Fixed scroll	Cast iron	19.46	0.24	0.13	0.13
Cross-slip ring	LY12	0.15	1.54×10^−3^	7.77×10^−4^	7.64×10^−4^
Small balance iron	Cast iron	0.22	5.57×10^−4^	2.83×10^−4^	2.76×10^−4^
Large balance iron	Cast iron	1.42	4.07×10^−3^	2.70×10^−3^	1.64×10^−3^
Belt pulley	HT200	2.77	7.92×10^−3^	4.31×10^−3^	4.31×10^−3^

### Flexible modeling

Compared to simulations involving rigid bodies, simulations with flexible bodies consider the dynamic stress and vibration deformation of the moving components, making them more accurate representations of the mechanical system’s bearing status [14]. As a result, dynamic simulations of flexible bodies are helpful for accurately calculating and analyzing micro-vibrations and micro-deformations in high-speed spindles, leading to more reliable simulation results.

**Fig 5:**
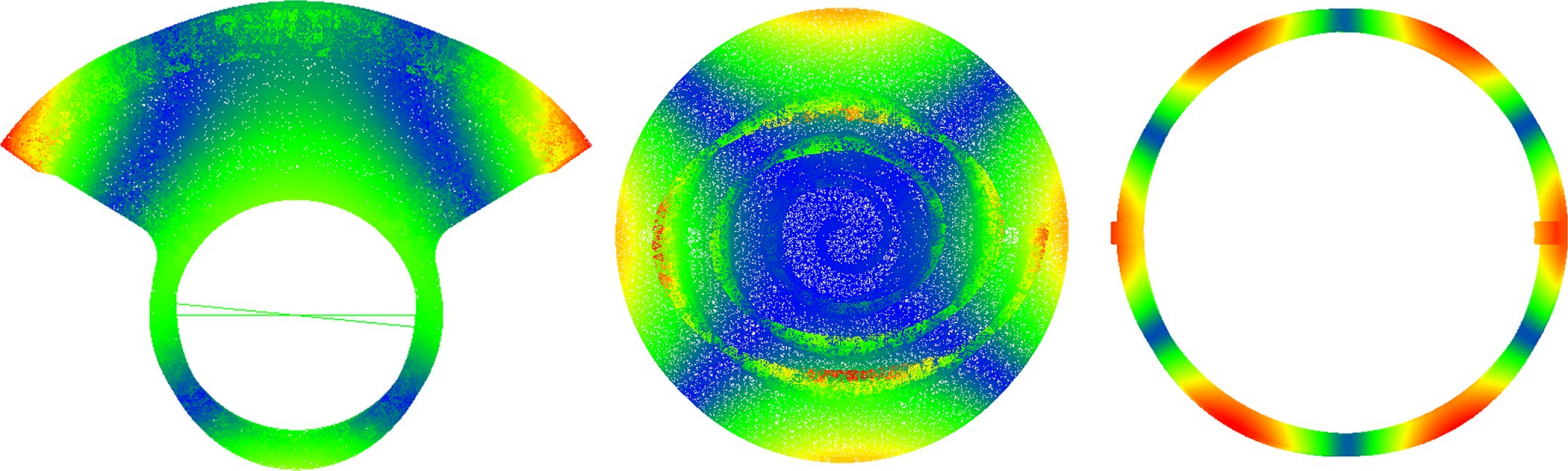
Flexible member included modal properties. (a) Large balance iron. (b) Orbiting scroll. (c) Cross-slip ring.

This paper utilizes the Viewflex module to build a flexible spindle model, which incorporates structural dispersion of the Solid tetrahedral entity element. The modeling data can be found in Table 3. With the help of a Modal Neutral File (MNF), the flexible body can be used to describe the elastic displacement of the moving component through a linear combination of modal vectors and modal coordinates [15], as illustrated in Fig 5. Based on this principle, a simulation model of the spindle’s rigid and flexible coupling is established by substituting the original rigid parts with flexible ones while maintaining the same motion constraint relationship.

**Table 3:** Modeling data of flexible components.

Component	Element type	Element size/mm	Element number	Node number
Crankshaft	Solid/Tetrahedral	0.5~2.3	85800	20278
Orbiting scroll	Solid/Tetrahedral	1.2~4.3	79071	20193
Cross-slip ring	Solid/Tetrahedral	0.7~1.8	47206	11762
Large balance iron	Solid/Tetrahedral	0.8~2.2	78843	17679
Small balance iron	Solid/Tetrahedral	0.6~1.8	31558	7925

**Fig 6:**
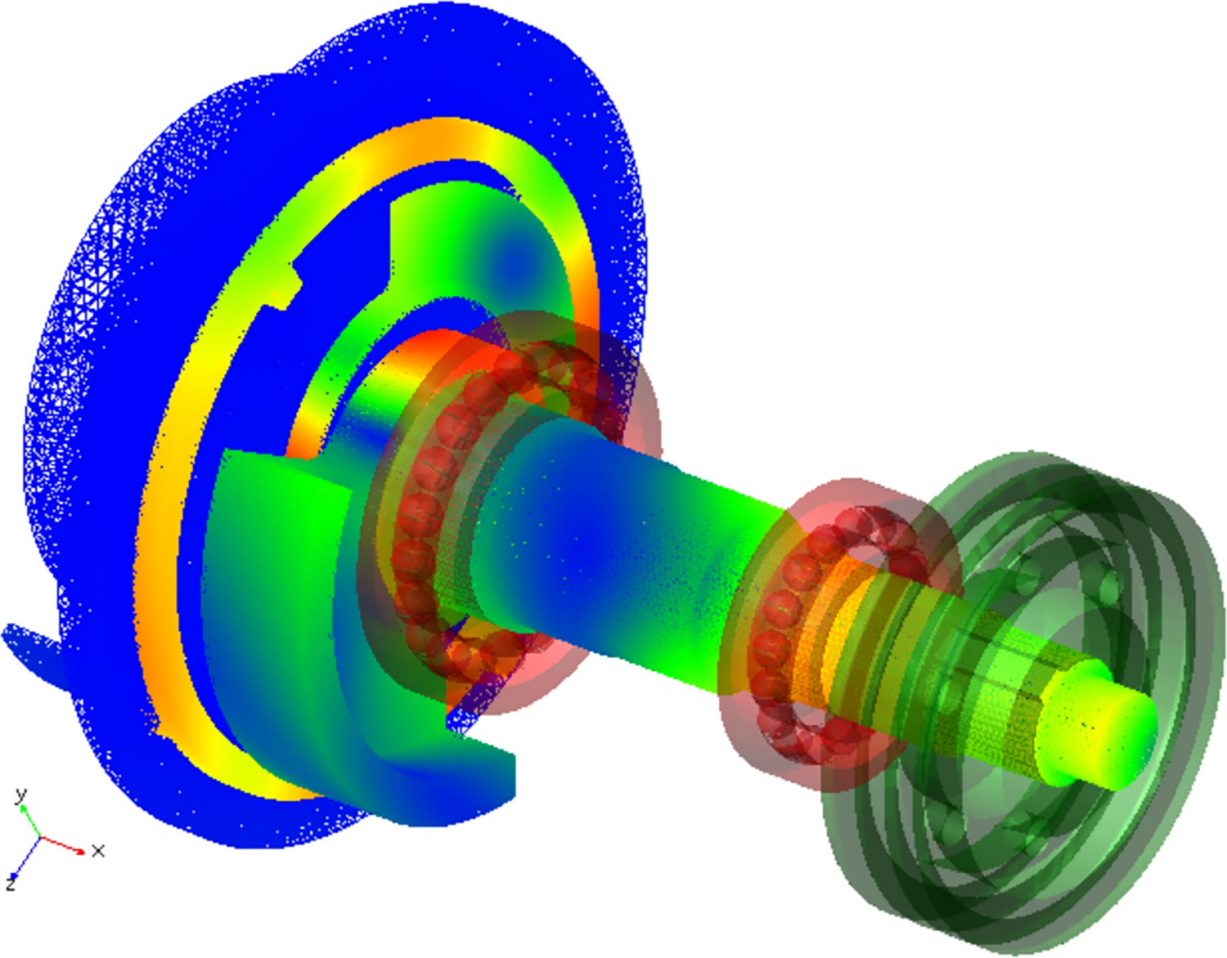
Flexible deformation of the spindle.

**Fig 7:**
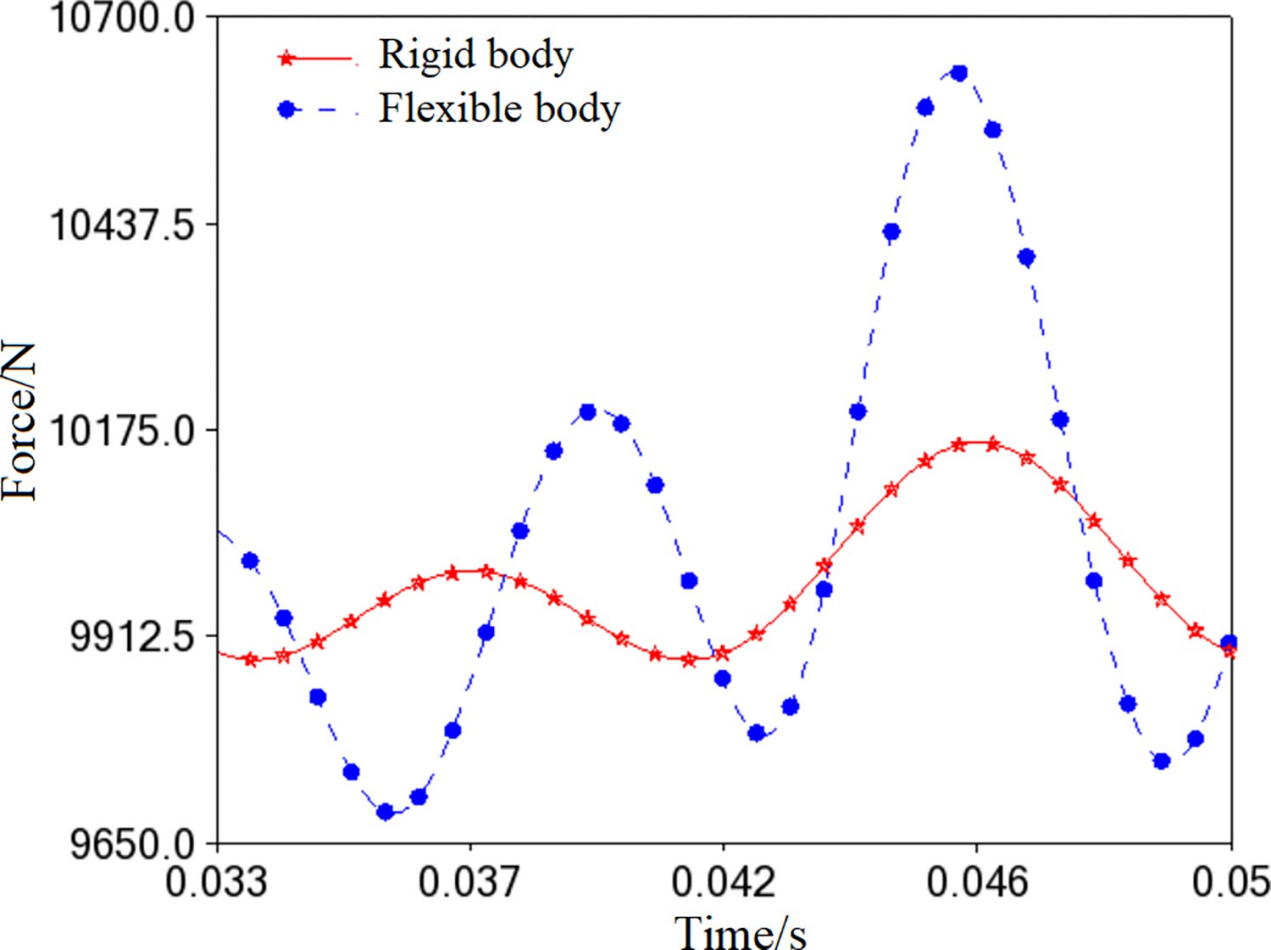
Comparison of simulation result.

The simulation of the flexible body is performed at a speed of 3600 r/min. The model is verified through static equilibrium analysis. Fig 6 shows the simulation of the flexible spindle system in real time. The stress and deformation of the flexible components are calculated in real-time, making the simulation more accurate and reliable [16]. As shown in Fig 7, the simulation curves for the needle-bearing constraint force are significantly different in the two states. The flexible body experiences more fluctuation due to the vibration deformation, indicating a greater constraint force inside the needle bearing. The results of simulating flexible bodies are helpful for accurately analyzing and understanding the high-speed running of the spindle.

## Flexible simulation with clearance

### Theoretical basis

#### Contact force

During high-speed operation of bearings, the rotating pair with clearance experiences alternating states of free motion and contact deformation. The contact collision force can be represented in a generalized Formula (6) [17]. Where: *F*_*n*_-normal contact force; *K*_n_-equivalent contact stiffness; *δ*-normal penetration depth; $\dot{\delta }$-normal relative collision velocity; *C*(*δ*)-damping factor associated with *δ*; *m*-force index (≥1).


$$F_{n}=K_{n}\delta^{m}+C(\delta )\dot{\delta }$$ (6)

#### Frictional force

It is crucial to consider the friction force that results from the contact between the crank pin and the bearing to understand any changes in the joint clearance correctly. We have adopted the modified Coulomb friction force model, illustrated in Formula (7) [18], to achieve this objective.


$$F_{t}=‐\mu (v_{t})F_{n}\frac{v_{t}}{\left|v_{t}\right|}$$ (7)

In Formula (7): *F*_*t*_-tangential friction force; *v*_t_-relative tangential sliding velocity at the contact point; *μ*(*v*_t_)-the sliding friction coefficient; *F*_*n*_-the normal contact force.

#### Impact function

ADAMS/View uses the Impact function to analyze contact and collision. Based on Hertz contact theory, this function considers both elastic and damping forces of the moving components. The Impact function uses a nonlinear spring-sampling model to simulate contact collision, improving contact calculations’ accuracy and reliability. When the value of the Impact function is 0 (*q*>*q*_1_), there is no contact between the moving components. On the other hand, if *q*≤*q*_1_, the Impact function is activated, indicating a collision between the moving components [19, 20].


$$\mathrm{Impact}=\{\begin{array}{l} 0\;q>q_{1}\\ k{\left(q_{1}-q\right)}^{e}-c_{\max}\dot{q}\cdot Step(q,q_{1}-d,1,q_{1},0)\;q\leq q_{1} \end{array}$$ (8)

In this formula, *k*-the contact stiffness coefficient (N/m); *q*-the measured displacement variable at the contact point (m); *q*_1_-the displacement threshold (m); *e*-force index (stiffness contribution factor); *c*_max_-the maximum damping coefficient (N·s/m); $\dot{q}$-the collision velocity at the contact point (m/s); *d*-penetration depth in maximum damping (m); *Step*-refers to the step function.

#### Boundary condition

**Fig 8:**
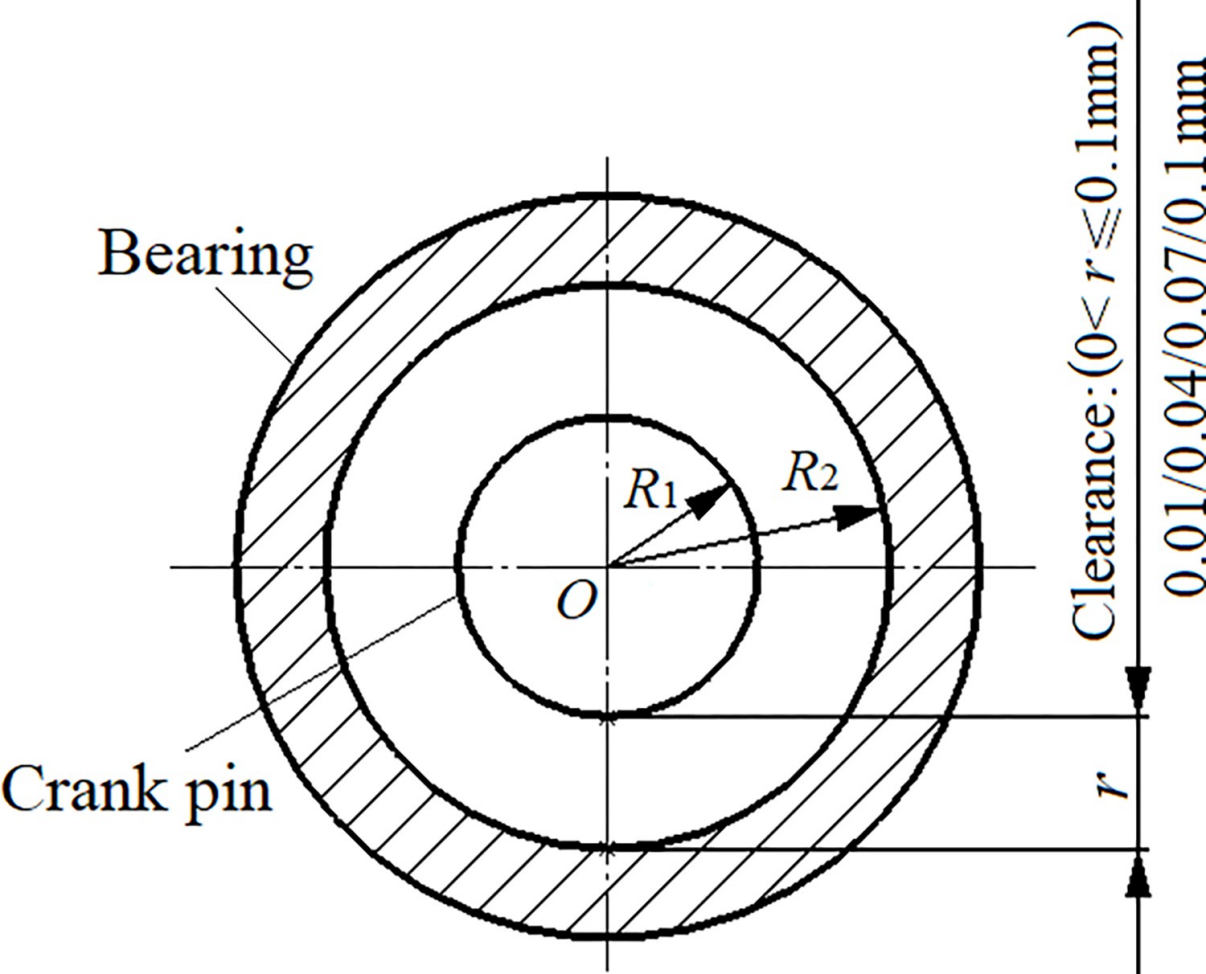
Rotating clearance.

In line with the machining accuracy and wear of the spindle, a contact clearance is established between the crankshaft and needle bearing, as depicted in Fig 8. The clearance values are 0.01mm, 0.04mm, 0.07mm, and 0.1mm, within the specified range of 0<*r*≤0.1mm. In the diagram, *R*_1_ denotes the crank pin radius, *R*_2_ represents the inner radius of the needle bearing, and *r* refers to the clearance radius (*r* = *R*_1_-*R*_2_).

**Fig 9:**
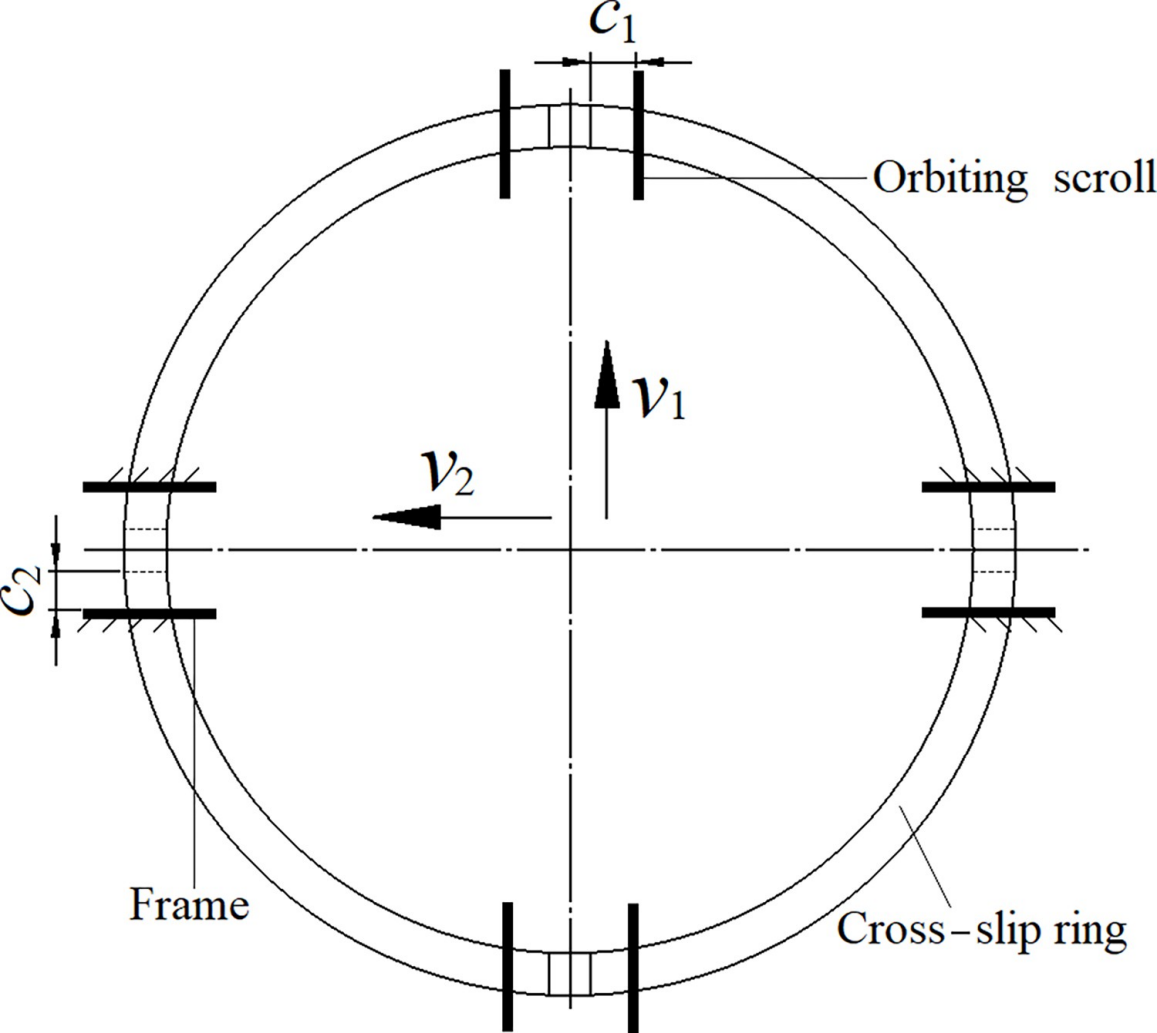
Translational clearance.

The translational pair consists of two clearances, *c*_1_, and *c*_2_, which are set between the slider and the orbiting scroll and between the slider and the frame. The *e*_*x*_ and *e*_*y*_ components refer to the center distance *o*_1_*o*_2_ in the *x*_1_ and *y*_1_ directions and are used to explore the anti-rotation effects of the cross-slip ring. In Fig 9, the cross-slip ring is shown to be translated in two vertical directions at velocities *v*_1_ and *v*_2_, respectively, when *c*_1_ and *c*_2_ are set to 0.07mm during the high-speed rotation of the spindle system. In the figure, *v*_1_ and *v*_2_ denote the translation speed of the cross-slip ring relative to the orbiting scroll and the frame, respectively, while *e*_*x*_ and *e*_*y*_ refer to the *x*_1_ and *y*_1_ components of the center distance *o*_1_*o*_2_.

The contact force and constraints between the moving components with clearance, such as the crank pin and needle scroll bearing (Flex Body to Flex Body), cross-slip ring & orbiting scroll (Flex Body to Flex Body), and cross-slip ring & frame (Flex Body to Solid), are defined in ADAMS/View using its Impact function and Coulomb friction function. The relevant parameters considered in this process include contact stiffness *K*, damping coefficient *C*, contact force index *e*, penetration depth *δ*_max_, dynamic friction factor *f*_d_, static friction factor *f*_s_, dynamic friction velocity *V*_d_, and static friction velocity *V*_s_, as listed in Table 4 [21].

**Table 4:** Parameters of contact force.

Parameters	*K*/N·m^-1^	*C*/N·s·m ^-1^	e	*δ*_max_/m	f_s_	f_d_	*V*_s_/m·s^-1^	*V*_d_/m·s^-1^
Value	1.0×10^8^	5.0×10^4^	1.5	1.0×10^−4^	0.08	0.05	1.0×10^−4^	1.0×10^−2^

#### Dynamic stress and deformation

**Fig 10:**
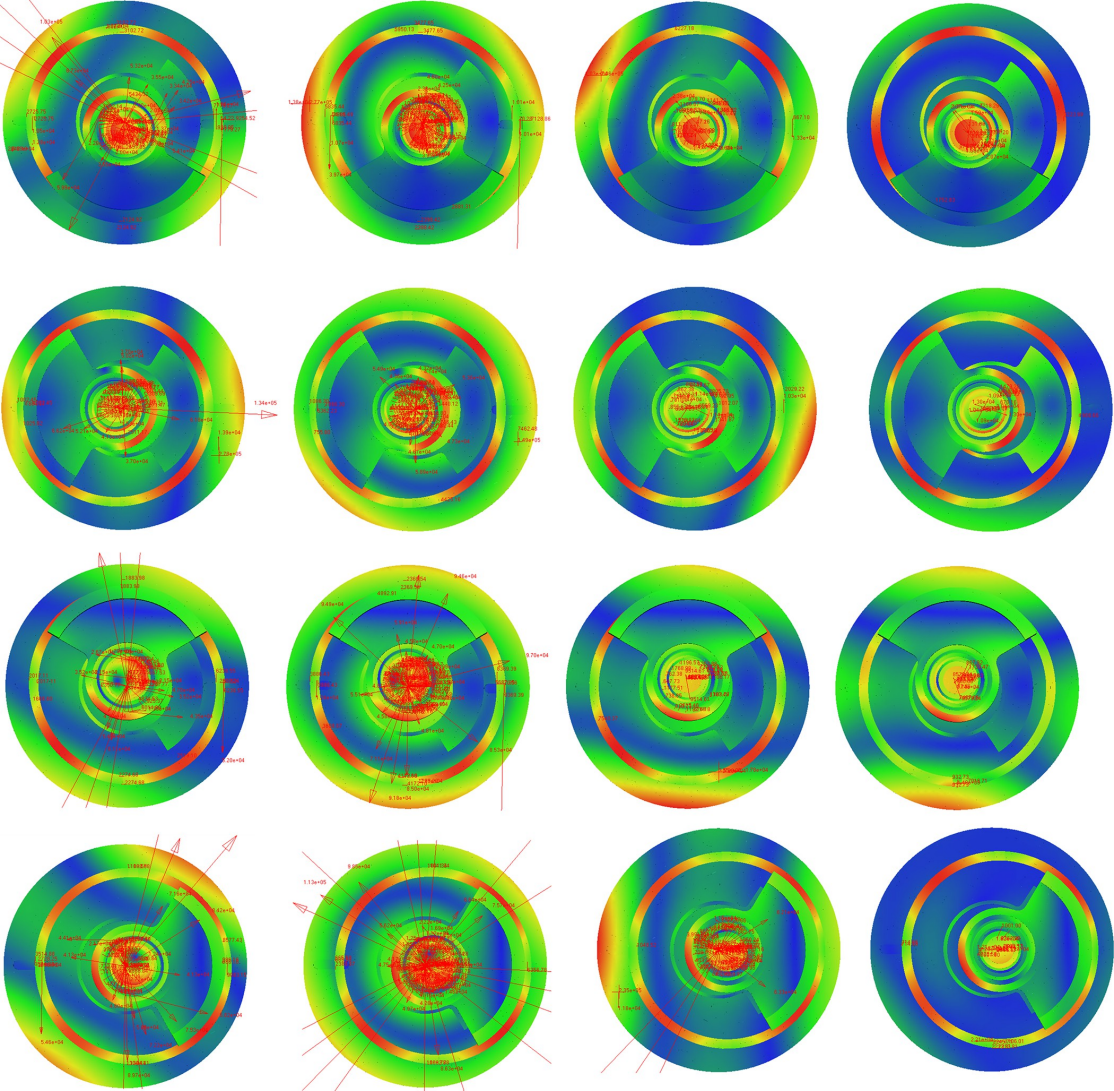
Flexible contact simulation in different clearances and positions. (a)*φ* = 90°, *r* = 0.01mm, (b) *φ* = 90°, *r* = 0.04mm, (c)*φ* = 90°, *r* = 0.07mm, (d)*φ* = 90°, *r* = 0.1mm, (e)*φ* = 180°, *r* = 0.01mm, (f)*φ* = 180°, *r* = 0.04mm, (g)*φ* = 180°, *r* = 0.07mm, (h)*φ* = 180°, *r* = 0.1mm, (i)*φ* = 270°, *r* = 0.01mm, (j)*φ* = 270°, *r* = 0.04mm, (k)*φ* = 270°, *r* = 0.07mm, (l) *φ* = 270°, *r* = 0.1mm, (m)*φ* = 360°, *r* = 0.01mm, (n)*φ* = 360°, *r* = 0.04mm, (o)*φ* = 360°, *r* = 0.07mm, (p)*φ* = 360°, *r* = 0.1mm.

The flexible spindle is dynamically simulated at 3600r/min for 0.1s, with 3200 steps taken for clearances of 0.01mm, 0.04mm, 0.07mm, and 0.1mm. As depicted in Fig 10, the instantaneous stress and vibration deformation of the spindle varies at different values of clearance when the crankshaft rotates 90°, 180°, 270°, and 360°.

In Fig 10, the parameter *φ* is crankshaft angle, *r* is joint clearance, and the red vector arrow represents the contact collision load of the moving component with clearance. The length of the arrow is proportional to the magnitude of the load. The real-time change in the simulated nephogram and the vector arrow helps to understand the contact and collision mechanism of the spindle system with clearance. This analysis is essential to understand the behavior of the spindle system under different clearance values.

## Results and discussion

### Ideal state

**Fig 11:**
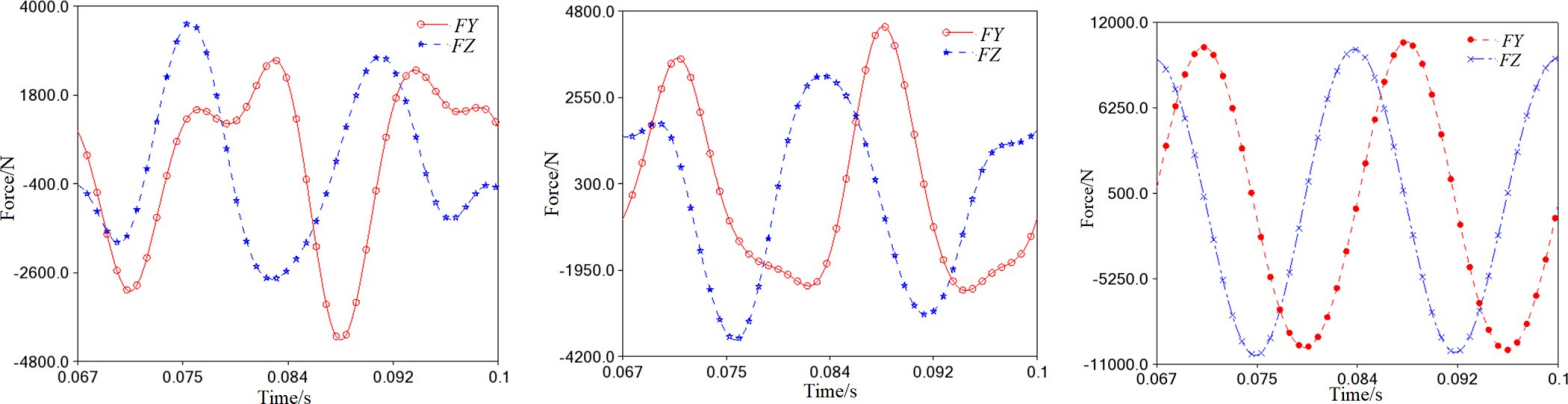
Support reactions of the bearings (*r* = 0mm). (a) Primary bearing. (b) Auxiliary bearing. c) Needle bearing.

In the ideal state (when *r* = 0mm), the impact of contact collision load can be disregarded. When the spindle operates with the *X*-axis as the rotary center, the support reaction of its internal bearing distributes in the *Y*-axis and *Z*-axis directions, as shown in Fig 11. The needle bearing experiences a significant internal support reaction compared to the primary and auxiliary bearings, indicating that most of the working load is applied to the crank pin. It is essential to ensure sufficient stiffness and strength reserves for the same. The support reaction curves are smooth and gentle, indicating that the running accuracy and stability of the spindle system will not be adversely affected. This is in line with the expected clearance in the ideal state.

**Fig 12:**
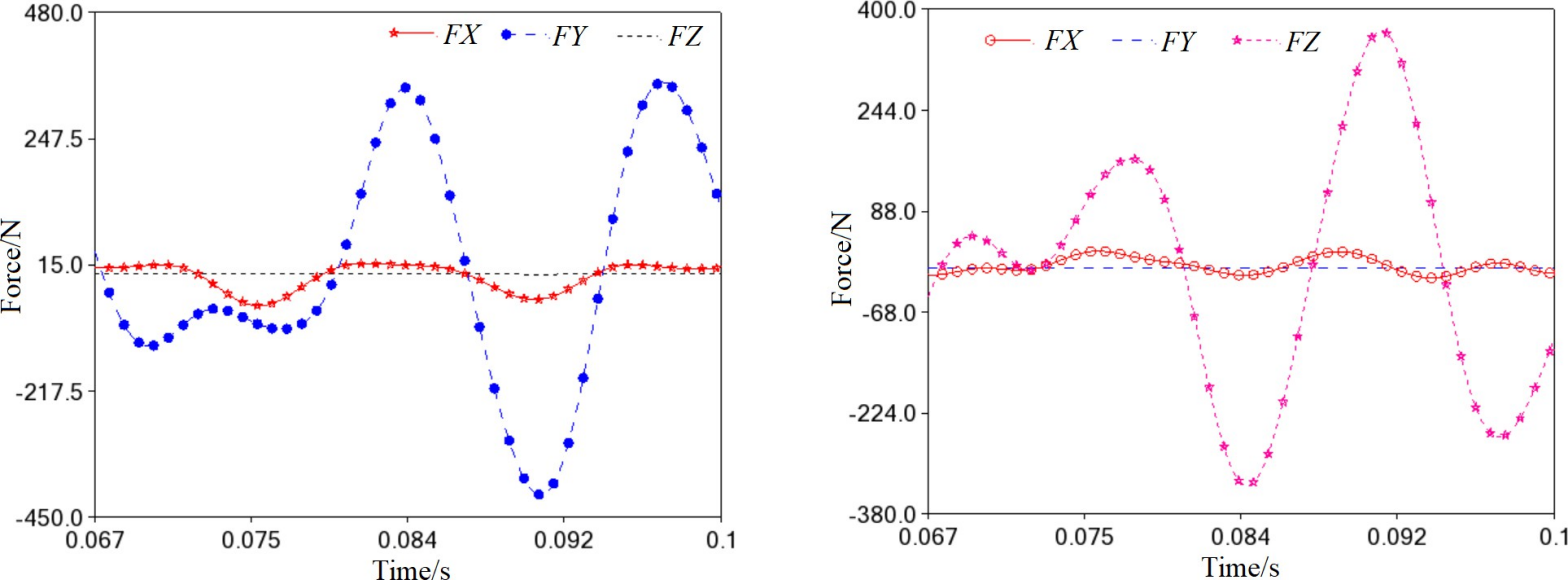
Constraint force of the cross-slip ring (*r* = 0mm). (a) Effect on the orbiting scroll. (b) Effect on the frame.

To ensure the scroll compressor’s proper functioning, the orbiting scroll must not rotate during inhalation, compression, and exhaust. For this reason, a limiting ring must be installed between the orbiting scroll and the frame, and the constraint force caused by the cross-slip ring in *X*, *Y*, and *Z* directions is illustrated in Fig 12(A) and 12(B), respectively. In the *Y*-axis direction, the constraint force between the cross-slip ring and the orbiting scroll dominates, while that between the cross-slip ring and the frame is in the *Z*-axis direction. The constraint force curves in both vertical directions are smooth and gentle, indicating that the cross-slip ring has an ideal anti-rotation effect.

#### Contact loads

**Fig 13:**
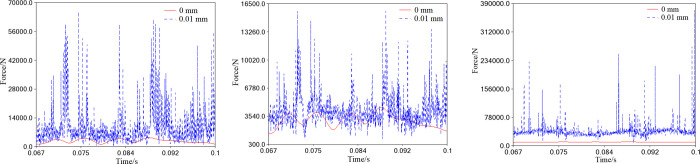
Support reactions of the bearings with clearance (*r* = 0.01mm). (a) Primary bearing. (b) Auxiliary bearing. (c) Needle bearing.

In Fig 13, we can see that the curve of the bearing support reaction with clearance is not as smooth as it should be in the ideal state. When the clearance of the bearing is 0.01mm, the reaction curves on the primary bearing, auxiliary bearing, and needle bearing fluctuate violently and change abruptly. This kind of clearance causes significant vibration impact and contact collision inside the bearing, leading to unstable spindle operation.

**Fig 14:**
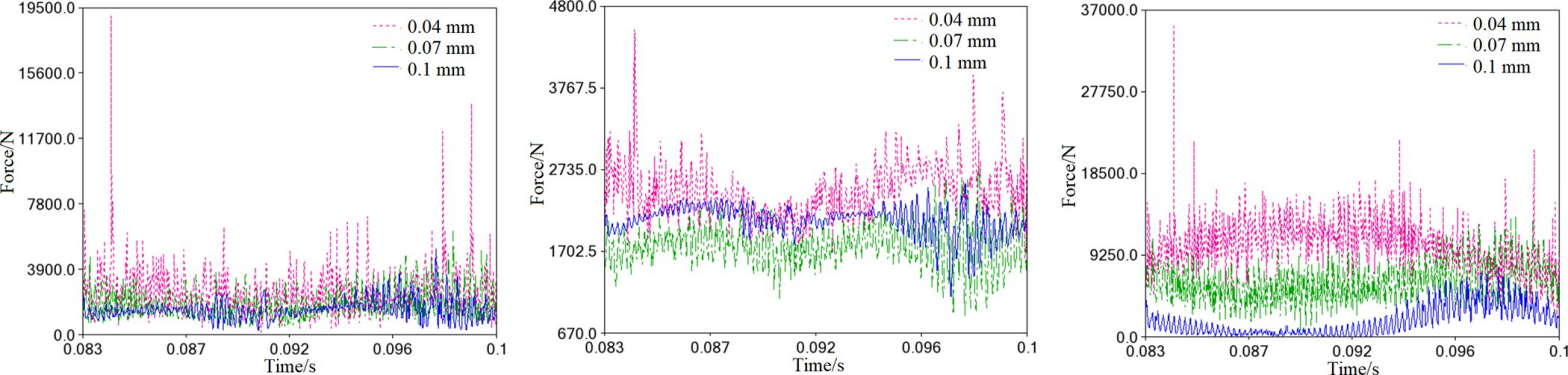
Support reactions of the bearings with clearance (0.01<*r*≤0.1mm). (a) Primary bearing. (b) Auxiliary bearing. (c) Needle bearing.

As shown in Fig 14, when the clearance increases to meet the requirement of 0.01<*r*≤0.1mm, the simulation curves of the support reaction are displayed. The comparative analysis suggests that the curve fluctuation range is much less than 0.01mm, indicating a significant decrease in the bearing support reaction with the clearance increase. Further comparison reveals no apparent differences in the size and fluctuation of the support reaction on the primary bearing among the three clearances, as demonstrated in Fig 14(A). For the auxiliary bearing, the support reaction increases from 0.07mm to 0.1mm to 0.04mm, as shown in Fig 14(B). Similarly, due to joint clearance, there is an unavoidable contact collision between the needle bearing and the crank pin, and the reaction force increases from 0.1mm to 0.07mm to 0.04mm, as depicted in Fig 14(C).

**Fig 15:**
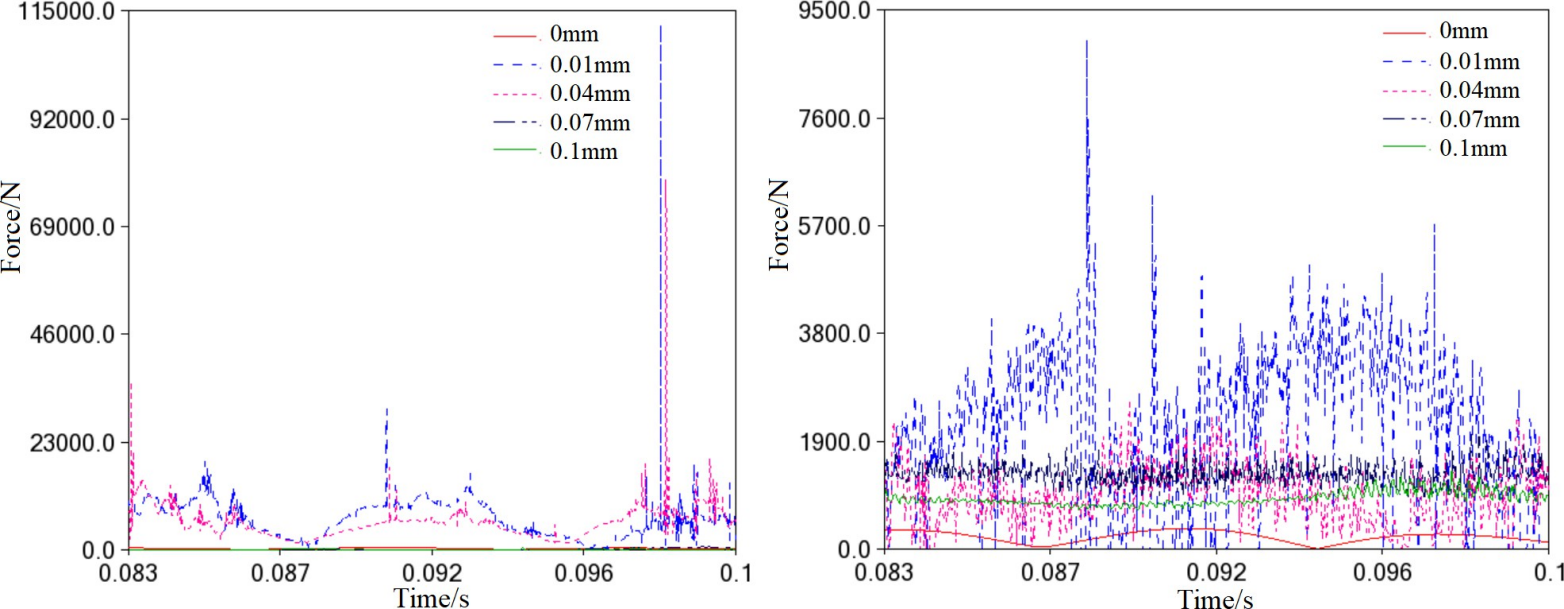
Contact load caused by the cross-slip ring (0<*r*≤0.1mm). (a) Impact on the orbiting scroll. (b) Impact on the frame.

During the translation process, the cross-slip ring will come into contact with the orbiting scroll and frame. The values *e*_*x*_ and *e*_*y*_ indicate the components of the center distance o_1_o_2_ in the *x*_1_ and *y*_1_ directions, respectively, as shown in Fig 15. The impact load between the ring and the orbiting scroll is particularly noticeable when the clearance is 0.01mm and 0.04mm (Fig 15(A)). However, when the clearance is increased to 0.07mm and 0.1mm, the impact load becomes negligible and can be ignored.

From the trend of the impact curves on the frame, it is evident that the force of collision between the ring and the frame considerably decreases to less than 9500N. However, the collision frequency intensifies, as Fig 15(B) depicts. The maximum collision force occurs at the clearance of 0.01mm. As the clearance increases to 0.01mm, the collision force flattens out.

#### Kinematic characteristics

**Fig 16:**
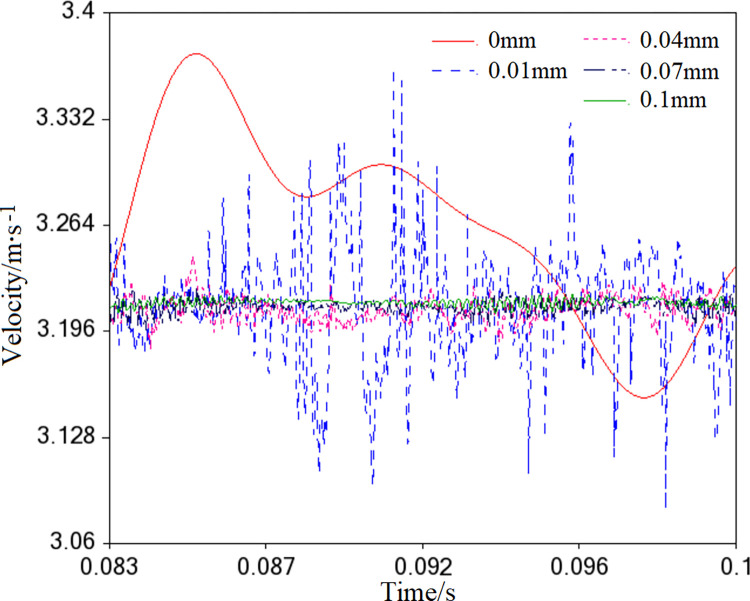
Velocity of the orbiting scroll.

Clearance inside the needle bearing can negatively impact the orbiting scroll’s circular path. Fig 16 illustrates that under different clearances, the velocity curve of the orbiting scroll deviates and oscillates compared to its ideal state (*r* = 0mm). The oscillation amplitude is more significant at a clearance of 0.01mm, indicating that the orbiting scroll’s motion suffers from significant shock and vibration, hindering the noise reduction of the scroll compressor. However, as the clearance continuously increases (e.g., 0.04mm, 0.07mm, and 0.1mm), the oscillation amplitude of the velocity curve decreases, which aligns with the expected contact load of the needle bearing with clearance.

**Fig 17:**
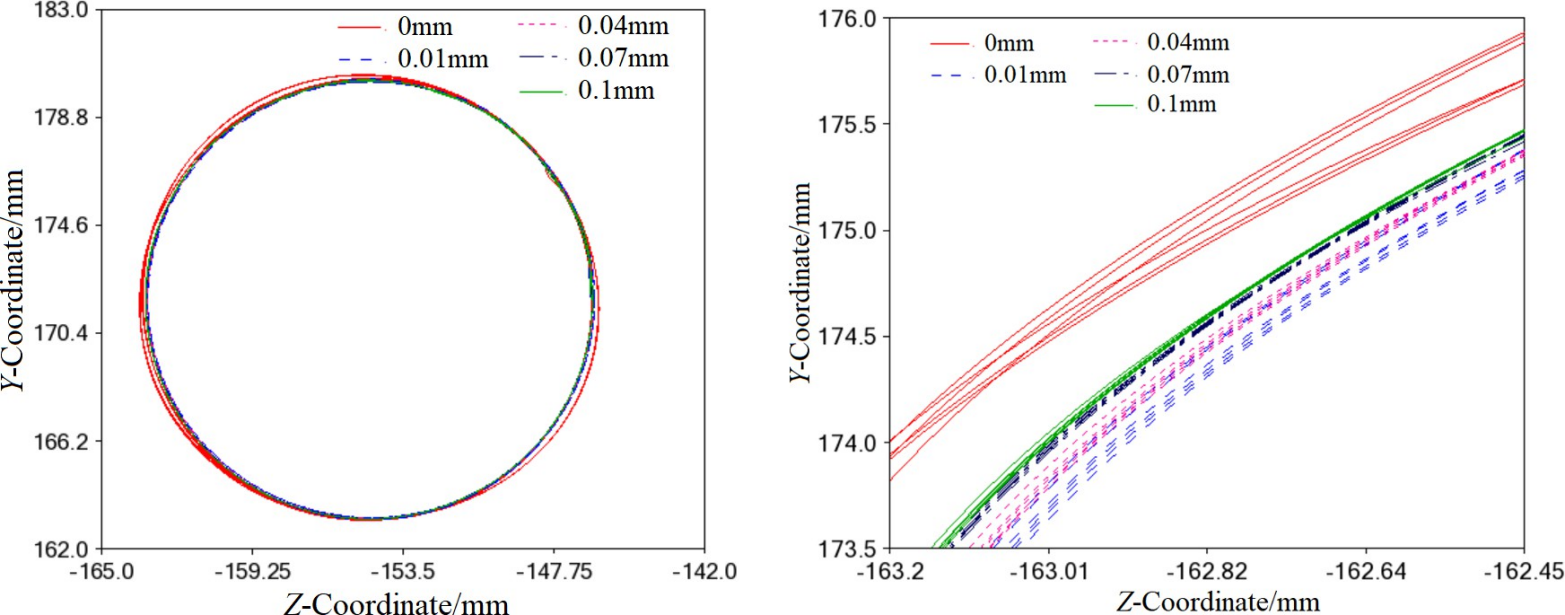
Centroid trajectory of the orbiting scroll. (a) Non-standard circular centroid trajectory. (b) Locally enlarged of the centroid trajectory.

In its ideal state, the Centroid trajectory of the orbiting scroll is a non-standard circle. However, in practice, the trajectory may shift or overlap to varying degrees, as shown in Fig 17 [22]. The figure clearly illustrates that the offset distance of the trajectory circle with clearance is 0.01mm, 0.04mm, 0.07mm, and 0.1mm from far to near successively. Therefore, the motion stationarity of the orbiting scroll is inversely related to the clearance size inside the needle bearing. The main reason is that an increase in the joint clearance reduces the friction resistance moment between the crank pin and the bearing. It is important to note that this phenomenon is restricted to the minimal joint clearance (0<*r*≤0.1mm).

#### Driving torque

**Fig 18:**
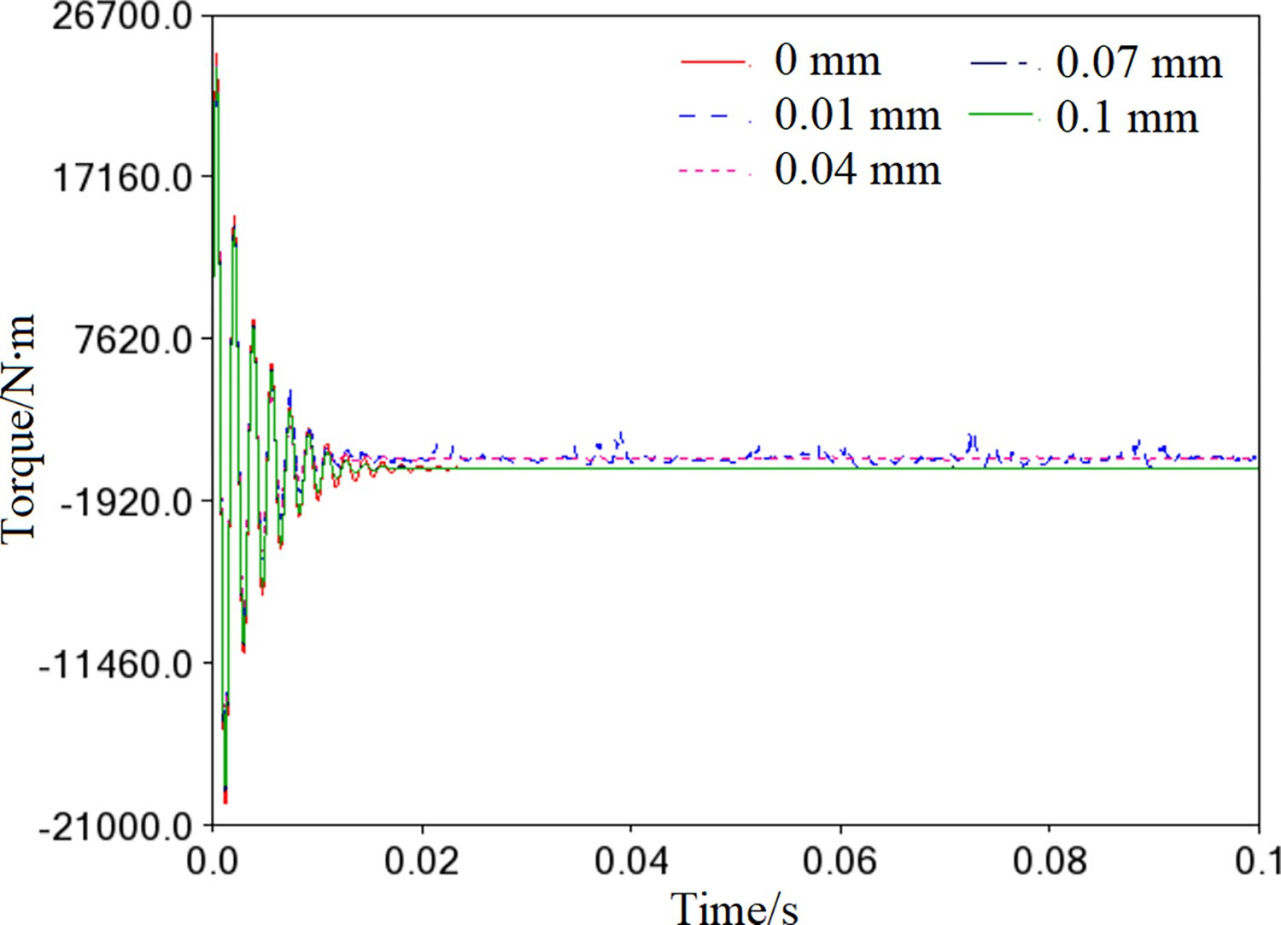
Motor torque curves in different clearance.

The scroll compressor may experience shock and vibration during the starting stage due to the motor’s unstable torque and inconsistent rotation. As shown in Fig 18, the spindle’s torque with clearance fluctuates erratically between 0~0.01mm, which is consistent with the actual starting situation of the motor. Although the torque curve gradually stabilizes over time, there is still considerable persistent oscillation and offset when the clearance is 0.01mm and 0.04mm compared to a clearance of 0mm. This indicates that the clearance of 0.01mm and 0.04mm significantly impact the spindle’s smooth operation.

## Conclusions and future work

This paper explores the impact of joint clearance on the scroll compressor through flexible modeling and dynamic simulation. Unlike previous studies, this approach offers a precise calculation and analysis of the micro-vibration and deformation of the spindle. The findings provide valuable insights for evaluating the performance of the scroll compressor with clearance.

Our team has carried out dynamic contact simulation of the critical flexible components like primary bearing, auxiliary bearing, needle bearing, orbiting scroll, and cross-slip ring under minimal clearance (0<*r*≤0.1mm). We have found that the spindle’s smooth operation is disrupted by significant collision loads when bearing clearance is between 0.01mm and 0.04mm. In contrast, the dynamic impact on the scroll compressor is relatively tiny in the clearance of 0.07mm and 0.1mm. It can be inferred that the vibration noise of the spindle is inversely proportional to the size of joint clearance.

When the clearance between components is at its ideal value (0mm), the orbiting scroll’s path of travel is an irregular circle with fluctuations. However, when the clearance between components increases, the flexible orbiting scroll experiences specific vibrations and deformations, causing its path of travel to overlap with the ideal path at multiple points. The offset distances from far to near are 0.01mm, 0.04mm, 0.07mm, and 0.1mm. This indicates that the smoothness of the orbiting scroll’s motion is inversely proportional to the size of the internal clearance of the needle roller bearing. Furthermore, the clearance in the spindle system can adversely affect the scroll compressor’s instantaneous start, especially when the clearance is 0.01mm and 0.04mm,which may lead to significant oscillation and deviation of the motor driving torque.

Compared to previous similar studies, this paper comprehensively considers the influence of the joint clearance on the spindle system based on flexible modeling, such as the clearance between the crank pin and the needle roller bearing, as well as the clearance at the orbiting scroll and the frame to the cross slip ring. On this basis, the slight vibration and deformation of the spindle system under high-speed conditions can be more accurately calculated and analyzed, which has a significant reference value to the performance evaluation of scroll compressor systems with clearance.

The effect of the clearance change is relatively easy to achieve in the computer environment, but it is challenging to involve specific experiments. Because the testing work is necessary to manufacture experimental samples of the spindle with clearances and also to build and debug the monitoring equipment. Besides, the cost and workload of the experiment are very massive, which is limited to the project research funds and actual conditions. For this reason, the experiment work cannot be carried out in a short time, and experimental research has yet to be found in similar work, while the simulation method is often used to predict the change of the joint clearance.

However, the experiment work is also a critical exploration direction that the authors care about. In the subsequent research, our team plans to focus on raising project funds and reforming the experimental site to make efforts and explore the experimental research to verify our work’s results and conclusions.

## Supporting information

S1 File Flexible and clearance simulation 0.1mm.part1. (RAR)

S2 File Flexible and clearance simulation 0.1mm.part2. (RAR)

S3 File Flexible and clearance simulation 0.1mm.part3. (RAR)

S4 File Flexible and clearance simulation 0.1mm.part4. (RAR)

S5 File Flexible and clearance simulation 0.1mm.part5. (RAR)
